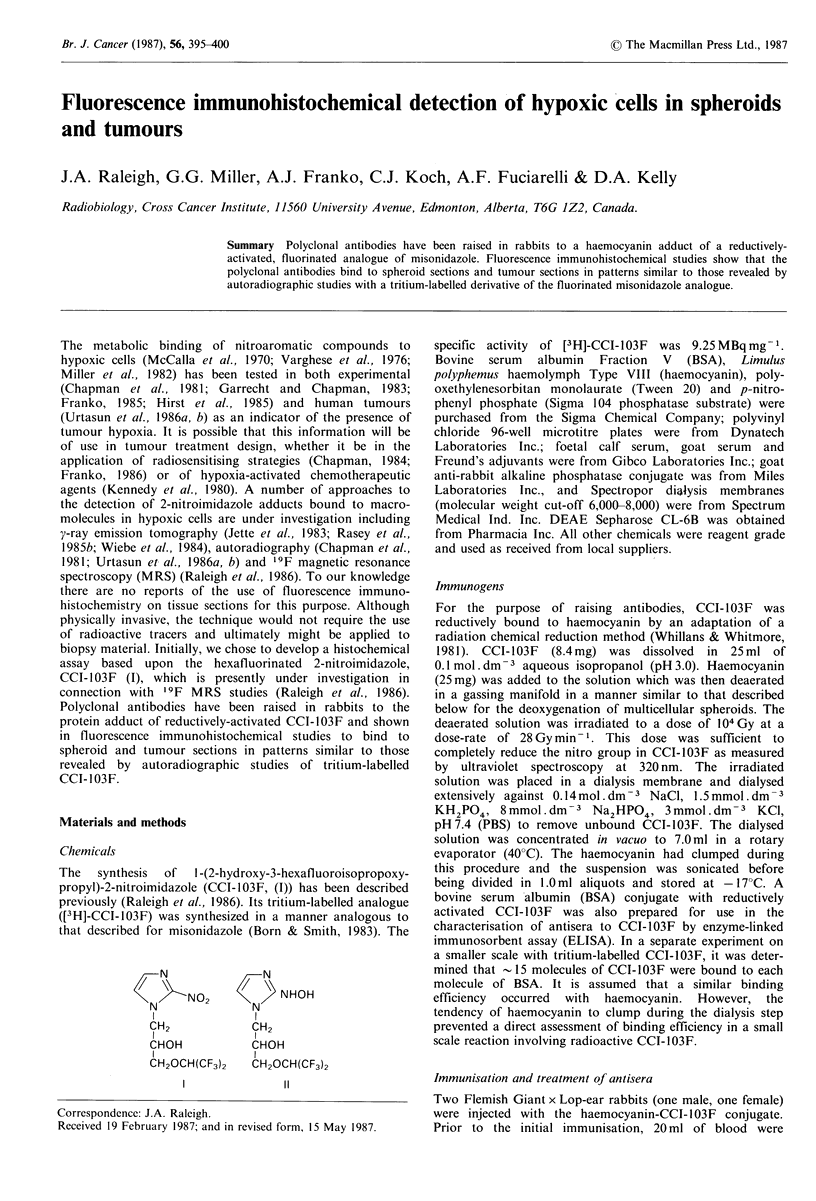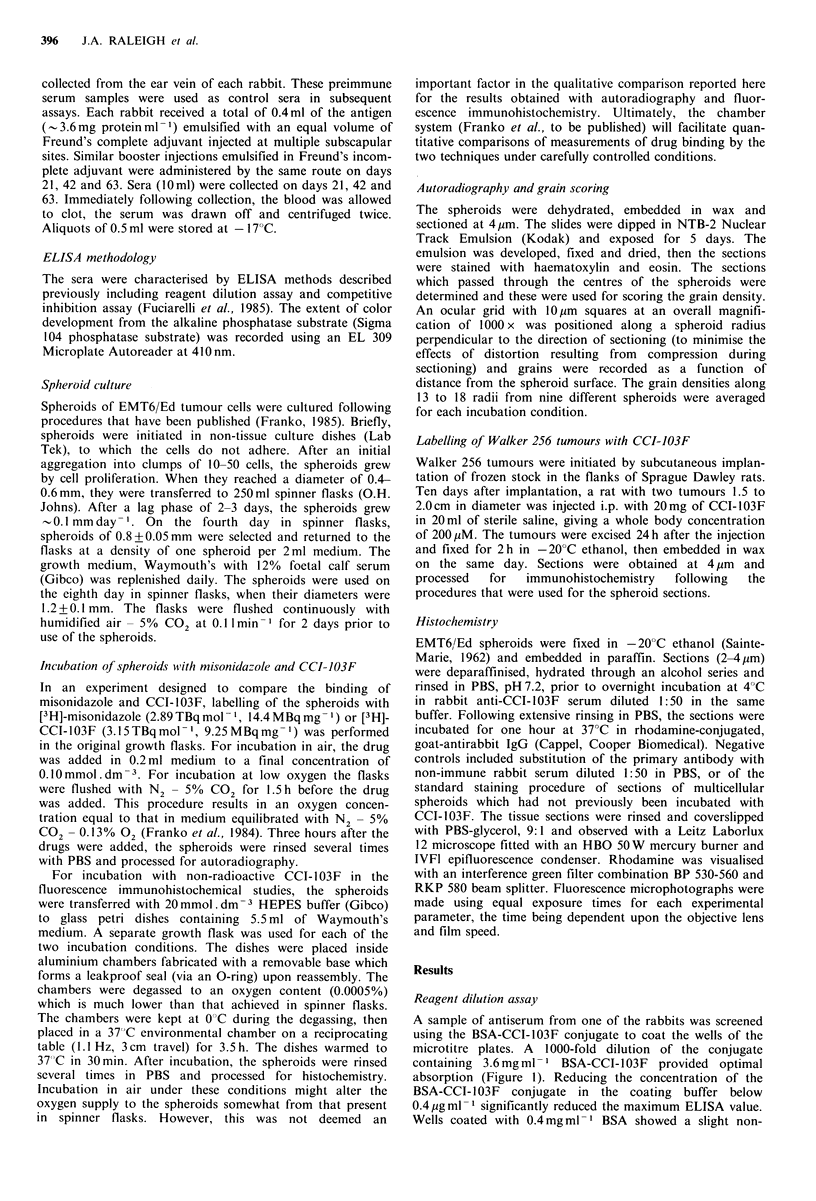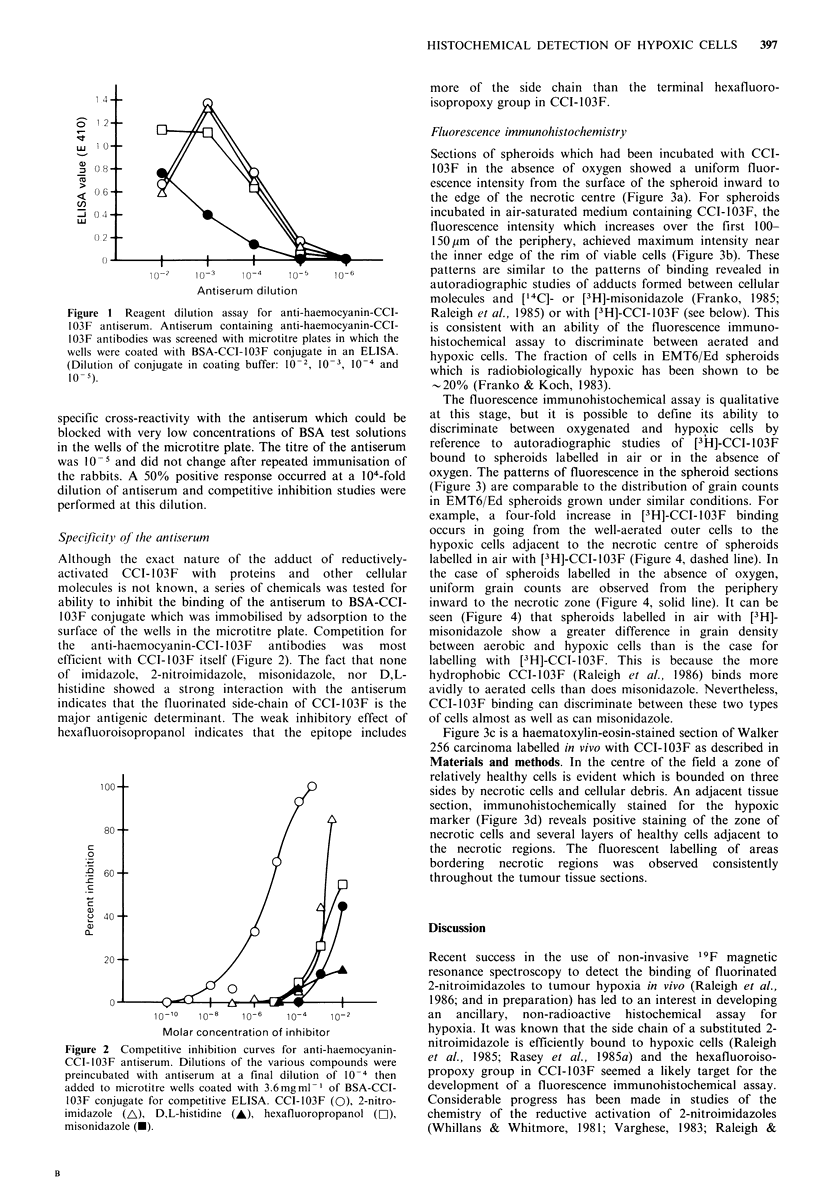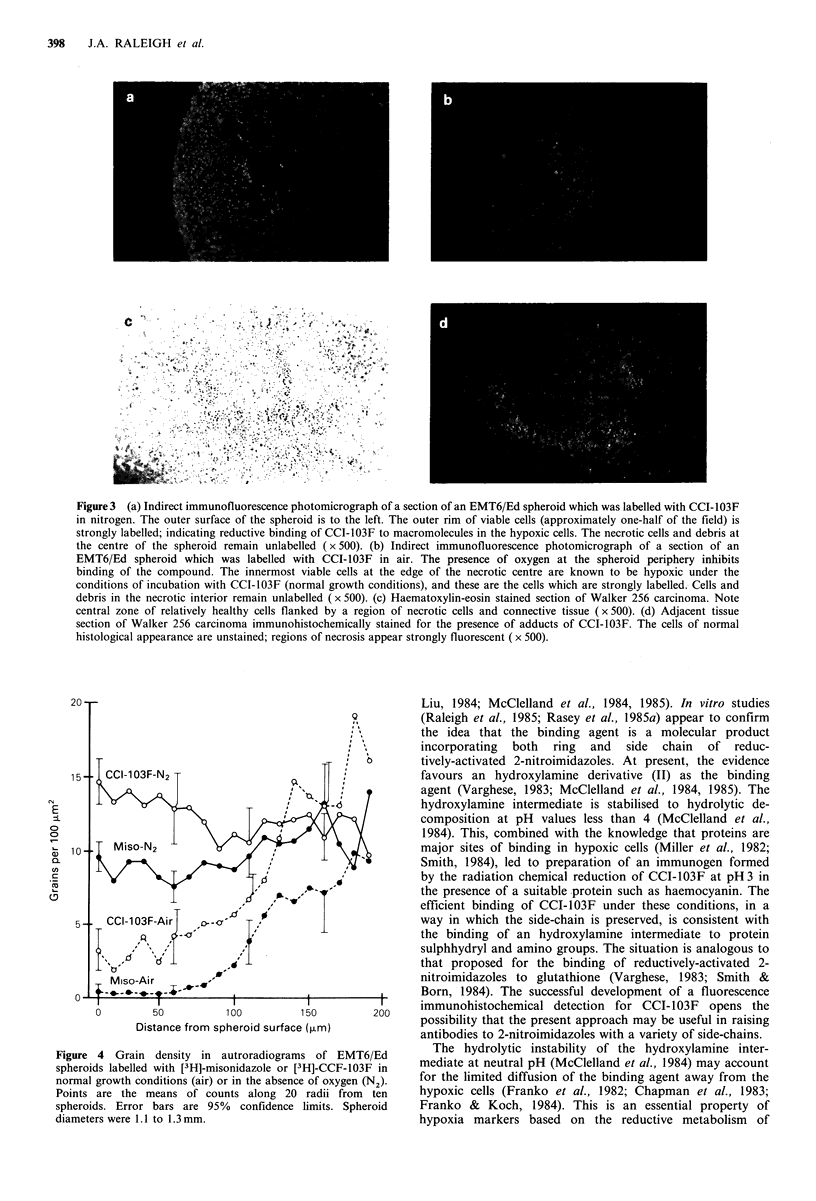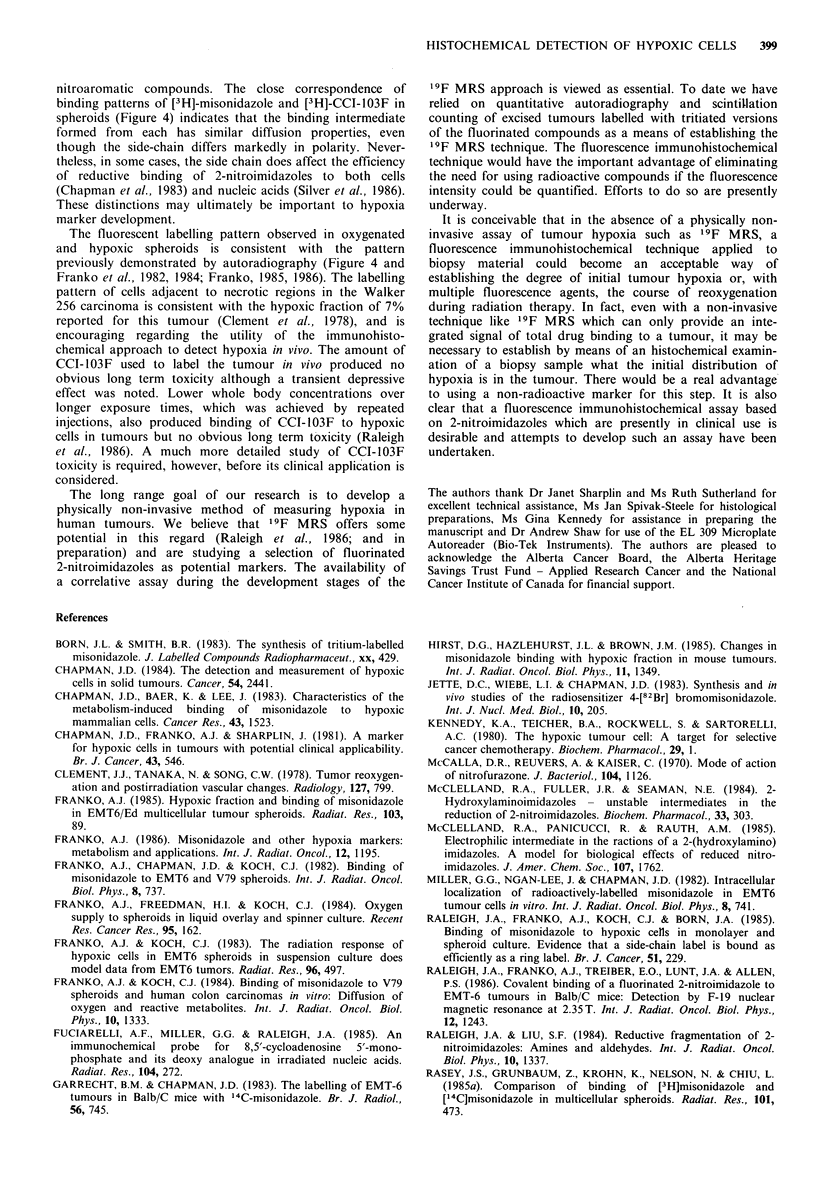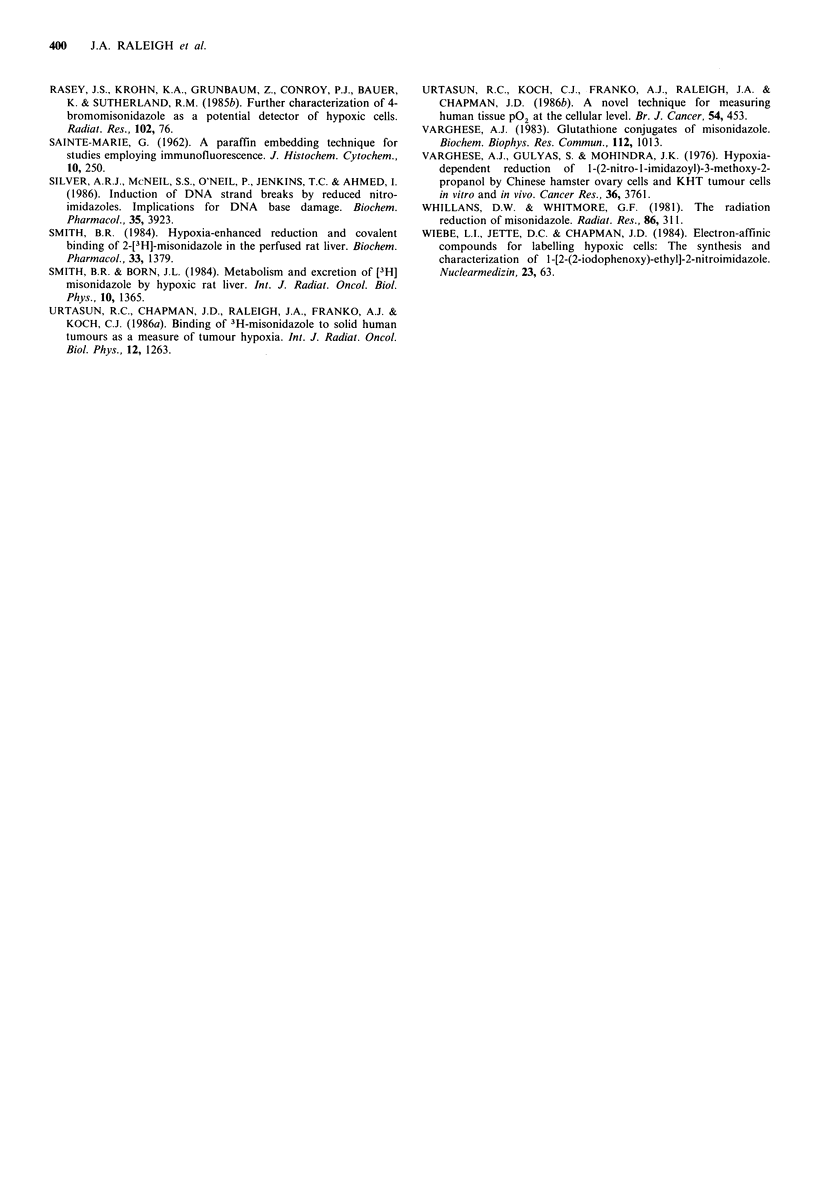# Fluorescence immunohistochemical detection of hypoxic cells in spheroids and tumours.

**DOI:** 10.1038/bjc.1987.213

**Published:** 1987-10

**Authors:** J. A. Raleigh, G. G. Miller, A. J. Franko, C. J. Koch, A. F. Fuciarelli, D. A. Kelly

**Affiliations:** Radiobiology, Cross Cancer Institute, Edmonton, Alberta, Canada.

## Abstract

**Images:**


*Br. J. Cancer* (1987), **56**, 395–400 © The Macmillan Press Ltd., 1987

### Fluorescence immunohistochemical detection of hypoxic cells in spheroids and tumours

J.A. Raleigh, G.G. Miller, A.J. Franko, C.J. Koch, A.F. Fuciarelli & D.A. Kelly

*Radiobiology, Cross Cancer Institute, 11560 University Avenue, Edmonton, Alberta, T6G 1Z2, Canada.*

**Summary** Polyclonal antibodies have been raised in rabbits to a haemocyanin adduct of a reductively-activated, fluorinated analogue of misonidazole. Fluorescence immunohistochemical studies show that the polyclonal antibodies bind to spheroid sections and tumour sections in patterns similar to those revealed by autoradiographic studies with a tritium-labelled derivative of the fluorinated misonidazole analogue.

The metabolic binding of nitroaromatic compounds to hypoxic cells (McCalla *et al.*, 1970; Varghese *et al.*, 1976; Miller *et al.*, 1982) has been tested in both experimental (Chapman *et al.*, 1981; Garrecht and Chapman, 1983; Franko, 1985; Hirst *et al.*, 1985) and human tumours (Urtasun *et al.*, 1986*a*, *b*) as an indicator of the presence of tumour hypoxia. It is possible that this information will be of use in tumour treatment design, whether it be in the application of radiosensitising strategies (Chapman, 1984; Franko, 1986) or of hypoxia-activated chemotherapeutic agents (Kennedy *et al.*, 1980). A number of approaches to the detection of 2-nitroimidazole adducts bound to macromolecules in hypoxic cells are under investigation including $\gamma$-ray emission tomography (Jette *et al.*, 1983; Rasey *et al.*, 1985*b*; Wiebe *et al.*, 1984), autoradiography (Chapman *et al.*, 1981; Urtasun *et al.*, 1986*a*, *b*) and $^{19}F$ magnetic resonance spectroscopy (MRS) (Raleigh *et al.*, 1986). To our knowledge there are no reports of the use of fluorescence immunohistochemistry on tissue sections for this purpose. Although physically invasive, the technique would not require the use of radioactive tracers and ultimately might be applied to biopsy material. Initially, we chose to develop a histochemical assay based upon the hexafluorinated 2-nitroimidazole, CCI-103F (I), which is presently under investigation in connection with $^{19}F$ MRS studies (Raleigh *et al.*, 1986). Polyclonal antibodies have been raised in rabbits to the protein adduct of reductively-activated CCI-103F and shown in fluorescence immunohistochemical studies to bind to spheroid and tumour sections in patterns similar to those revealed by autoradiographic studies of tritium-labelled CCI-103F.

### Materials and methods

### *Chemicals*

The synthesis of 1-(2-hydroxy-3-hexafluoroisopropoxy-propyl)-2-nitroimidazole (CCI-103F, (I)) has been described previously (Raleigh *et al.*, 1986). Its tritium-labelled analogue ([$^3$H]-CCI-103F) was synthesized in a manner analogous to that described for misonidazole (Born & Smith, 1983). The

I

II

Correspondence: J.A. Raleigh.
Received 19 February 1987; and in revised form, 15 May 1987.

specific activity of [$^3$H]-CCI-103F was 9.25 MBq mg$^{-1}$. Bovine serum albumin Fraction V (BSA), *Limulus polyphemus* haemolymph Type VIII (haemocyanin), polyoxethylenesorbitan monolaurate (Tween 20) and *p*-nitrophenyl phosphate (Sigma 104 phosphatase substrate) were purchased from the Sigma Chemical Company; polyvinyl chloride 96-well microtitre plates were from Dynatech Laboratories Inc.; foetal calf serum, goat serum and Freund's adjuvants were from Gibco Laboratories Inc.; goat anti-rabbit alkaline phosphatase conjugate was from Miles Laboratories Inc., and Spectropor dialysis membranes (molecular weight cut-off 6,000–8,000) were from Spectrum Medical Ind. Inc. DEAE Sepharose CL-6B was obtained from Pharmacia Inc. All other chemicals were reagent grade and used as received from local suppliers.

### *Immunogens*

For the purpose of raising antibodies, CCI-103F was reductively bound to haemocyanin by an adaptation of a radiation chemical reduction method (Whillans & Whitmore, 1981). CCI-103F (8.4 mg) was dissolved in 25 ml of 0.1 mol.dm$^{-3}$ aqueous isopropanol (pH 3.0). Haemocyanin (25 mg) was added to the solution which was then deaerated in a gassing manifold in a manner similar to that described below for the deoxygenation of multicellular spheroids. The deaerated solution was irradiated to a dose of $10^4$ Gy at a dose-rate of 28 Gy min$^{-1}$. This dose was sufficient to completely reduce the nitro group in CCI-103F as measured by ultraviolet spectroscopy at 320 nm. The irradiated solution was placed in a dialysis membrane and dialysed extensively against 0.14 mol.dm$^{-3}$ NaCl, 1.5 mmol.dm$^{-3}$ $KH_2PO_4$, 8 mmol.dm$^{-3}$ $Na_2HPO_4$, 3 mmol.dm$^{-3}$ KCl, pH 7.4 (PBS) to remove unbound CCI-103F. The dialysed solution was concentrated *in vacuo* to 7.0 ml in a rotary evaporator (40°C). The haemocyanin had clumped during this procedure and the suspension was sonicated before being divided in 1.0 ml aliquots and stored at −17°C. A bovine serum albumin (BSA) conjugate with reductively activated CCI-103F was also prepared for use in the characterisation of antisera to CCI-103F by enzyme-linked immunosorbent assay (ELISA). In a separate experiment on a smaller scale with tritium-labelled CCI-103F, it was determined that ~15 molecules of CCI-103F were bound to each molecule of BSA. It is assumed that a similar binding efficiency occurred with haemocyanin. However, the tendency of haemocyanin to clump during the dialysis step prevented a direct assessment of binding efficiency in a small scale reaction involving radioactive CCI-103F.

### *Immunisation and treatment of antisera*

Two Flemish Giant × Lop-ear rabbits (one male, one female) were injected with the haemocyanin-CCI-103F conjugate. Prior to the initial immunisation, 20 ml of blood were collected from the ear vein of each rabbit. These preimmune serum samples were used as control sera in subsequent assays. Each rabbit received a total of 0.4 ml of the antigen ($\sim$3.6 mg protein ml$^{-1}$) emulsified with an equal volume of Freund's complete adjuvant injected at multiple subscapular sites. Similar booster injections emulsified in Freund's incomplete adjuvant were administered by the same route on days 21, 42 and 63. Sera (10 ml) were collected on days 21, 42 and 63. Immediately following collection, the blood was allowed to clot, the serum was drawn off and centrifuged twice. Aliquots of 0.5 ml were stored at $-17$°C.

*ELISA methodology*

The sera were characterised by ELISA methods described previously including reagent dilution assay and competitive inhibition assay (Fuciarelli et al., 1985). The extent of color development from the alkaline phosphatase substrate (Sigma 104 phosphatase substrate) was recorded using an EL 309 Microplate Autoreader at 410 nm.

*Spheroid culture*

Spheroids of EMT6/Ed tumour cells were cultured following procedures that have been published (Franko, 1985). Briefly, spheroids were initiated in non-tissue culture dishes (Lab Tek), to which the cells do not adhere. After an initial aggregation into clumps of 10–50 cells, the spheroids grew by cell proliferation. When they reached a diameter of 0.4–0.6 mm, they were transferred to 250 ml spinner flasks (O.H. Johns). After a lag phase of 2–3 days, the spheroids grew $\sim$0.1 mm day$^{-1}$. On the fourth day in spinner flasks, spheroids of $0.8 \pm 0.05$ mm were selected and returned to the flasks at a density of one spheroid per 2 ml medium. The growth medium, Waymouth's with 12% foetal calf serum (Gibco) was replenished daily. The spheroids were used on the eighth day in spinner flasks, when their diameters were $1.2 \pm 0.1$ mm. The flasks were flushed continuously with humidified air – 5% $CO_2$ at 0.1 l min$^{-1}$ for 2 days prior to use of the spheroids.

*Incubation of spheroids with misonidazole and CCI-103F*

In an experiment designed to compare the binding of misonidazole and CCI-103F, labelling of the spheroids with [$^3$H]-misonidazole (2.89 TBq mol$^{-1}$, 14.4 MBq mg$^{-1}$) or [$^3$H]-CCI-103F (3.15 TBq mol$^{-1}$, 9.25 MBq mg$^{-1}$) was performed in the original growth flasks. For incubation in air, the drug was added in 0.2 ml medium to a final concentration of 0.10 mmol.dm$^{-3}$. For incubation at low oxygen the flasks were flushed with $N_2$ – 5% $CO_2$ for 1.5 h before the drug was added. This procedure results in an oxygen concentration equal to that in medium equilibrated with $N_2$ – 5% $CO_2$ – 0.13% $O_2$ (Franko et al., 1984). Three hours after the drugs were added, the spheroids were rinsed several times with PBS and processed for autoradiography.

For incubation with non-radioactive CCI-103F in the fluorescence immunohistochemical studies, the spheroids were transferred with 20 mmol.dm$^{-3}$ HEPES buffer (Gibco) to glass petri dishes containing 5.5 ml of Waymouth's medium. A separate growth flask was used for each of the two incubation conditions. The dishes were placed inside aluminium chambers fabricated with a removable base which forms a leakproof seal (via an O-ring) upon reassembly. The chambers were degassed to an oxygen content (0.0005%) which is much lower than that achieved in spinner flasks. The chambers were kept at 0°C during the degassing, then placed in a 37°C environmental chamber on a reciprocating table (1.1 Hz, 3 cm travel) for 3.5 h. The dishes warmed to 37°C in 30 min. After incubation, the spheroids were rinsed several times in PBS and processed for histochemistry. Incubation in air under these conditions might alter the oxygen supply to the spheroids somewhat from that present in spinner flasks. However, this was not deemed an important factor in the qualitative comparison reported here for the results obtained with autoradiography and fluorescence immunohistochemistry. Ultimately, the chamber system (Franko et al., to be published) will facilitate quantitative comparisons of measurements of drug binding by the two techniques under carefully controlled conditions.

*Autoradiography and grain scoring*

The spheroids were dehydrated, embedded in wax and sectioned at 4 $\mu$m. The slides were dipped in NTB-2 Nuclear Track Emulsion (Kodak) and exposed for 5 days. The emulsion was developed, fixed and dried, then the sections were stained with haematoxylin and eosin. The sections which passed through the centres of the spheroids were determined and these were used for scoring the grain density. An ocular grid with 10 $\mu$m squares at an overall magnification of 1000 × was positioned along a spheroid radius perpendicular to the direction of sectioning (to minimise the effects of distortion resulting from compression during sectioning) and grains were recorded as a function of distance from the spheroid surface. The grain densities along 13 to 18 radii from nine different spheroids were averaged for each incubation condition.

*Labelling of Walker 256 tumours with CCI-103F*

Walker 256 tumours were initiated by subcutaneous implantation of frozen stock in the flanks of Sprague Dawley rats. Ten days after implantation, a rat with two tumours 1.5 to 2.0 cm in diameter was injected i.p. with 20 mg of CCI-103F in 20 ml of sterile saline, giving a whole body concentration of 200 $\mu$M. The tumours were excised 24 h after the injection and fixed for 2 h in $-20$°C ethanol, then embedded in wax on the same day. Sections were obtained at 4 $\mu$m and processed for immunohistochemistry following the procedures that were used for the spheroid sections.

*Histochemistry*

EMT6/Ed spheroids were fixed in $-20$°C ethanol (Sainte-Marie, 1962) and embedded in paraffin. Sections (2–4 $\mu$m) were deparaffinised, hydrated through an alcohol series and rinsed in PBS, pH 7.2, prior to overnight incubation at 4°C in rabbit anti-CCI-103F serum diluted 1:50 in the same buffer. Following extensive rinsing in PBS, the sections were incubated for one hour at 37°C in rhodamine-conjugated, goat-antirabbit IgG (Cappel, Cooper Biomedical). Negative controls included substitution of the primary antibody with non-immune rabbit serum diluted 1:50 in PBS, or of the standard staining procedure of sections of multicellular spheroids which had not previously been incubated with CCI-103F. The tissue sections were rinsed and coverslipped with PBS-glycerol, 9:1 and observed with a Leitz Laborlux 12 microscope fitted with an HBO 50 W mercury burner and IVFl epifluorescence condenser. Rhodamine was visualised with an interference green filter combination BP 530-560 and RKP 580 beam splitter. Fluorescence microphotographs were made using equal exposure times for each experimental parameter, the time being dependent upon the objective lens and film speed.

### Results

*Reagent dilution assay*

A sample of antiserum from one of the rabbits was screened using the BSA-CCI-103F conjugate to coat the wells of the microtitre plates. A 1000-fold dilution of the conjugate containing 3.6 mg ml$^{-1}$ BSA-CCI-103F provided optimal absorption (Figure 1). Reducing the concentration of the BSA-CCI-103F conjugate in the coating buffer below 0.4 $\mu$g ml$^{-1}$ significantly reduced the maximum ELISA value. Wells coated with 0.4 mg ml$^{-1}$ BSA showed a slight non-

**Figure 1** Reagent dilution assay for anti-haemocyanin-CCI-103F antiserum. Antiserum containing anti-haemocyanin-CCI-103F antibodies was screened with microtitre plates in which the wells were coated with BSA-CCI-103F conjugate in an ELISA. (Dilution of conjugate in coating buffer: $10^{-2}$, $10^{-3}$, $10^{-4}$ and $10^{-5}$).

specific cross-reactivity with the antiserum which could be blocked with very low concentrations of BSA test solutions in the wells of the microtitre plate. The titre of the antiserum was $10^{-5}$ and did not change after repeated immunisation of the rabbits. A 50% positive response occurred at a $10^4$-fold dilution of antiserum and competitive inhibition studies were performed at this dilution.

*Specificity of the antiserum*

Although the exact nature of the adduct of reductively-activated CCI-103F with proteins and other cellular molecules is not known, a series of chemicals was tested for ability to inhibit the binding of the antiserum to BSA-CCI-103F conjugate which was immobilised by adsorption to the surface of the wells in the microtitre plate. Competition for the anti-haemocyanin-CCI-103F antibodies was most efficient with CCI-103F itself (Figure 2). The fact that none of imidazole, 2-nitroimidazole, misonidazole, nor D,L-histidine showed a strong interaction with the antiserum indicates that the fluorinated side-chain of CCI-103F is the major antigenic determinant. The weak inhibitory effect of hexafluoroisopropanol indicates that the epitope includes more of the side chain than the terminal hexafluoroisopropoxy group in CCI-103F.

**Figure 2** Competitive inhibition curves for anti-haemocyanin-CCI-103F antiserum. Dilutions of the various compounds were preincubated with antiserum at a final dilution of $10^{-4}$ then added to microtitre wells coated with $3.6\,mg\,ml^{-1}$ of BSA-CCI-103F conjugate for competitive ELISA. CCI-103F (○), 2-nitroimidazole (△), D,L-histidine (▲), hexafluoropropanol (□), misonidazole (■).

*Fluorescence immunohistochemistry*

Sections of spheroids which had been incubated with CCI-103F in the absence of oxygen showed a uniform fluorescence intensity from the surface of the spheroid inward to the edge of the necrotic centre (Figure 3a). For spheroids incubated in air-saturated medium containing CCI-103F, the fluorescence intensity which increases over the first 100–150 $\mu$m of the periphery, achieved maximum intensity near the inner edge of the rim of viable cells (Figure 3b). These patterns are similar to the patterns of binding revealed in autoradiographic studies of adducts formed between cellular molecules and [$^{14}$C]- or [$^3$H]-misonidazole (Franko, 1985; Raleigh *et al.*, 1985) or with [$^3$H]-CCI-103F (see below). This is consistent with an ability of the fluorescence immunohistochemical assay to discriminate between aerated and hypoxic cells. The fraction of cells in EMT6/Ed spheroids which is radiobiologically hypoxic has been shown to be ~20% (Franko & Koch, 1983).

The fluorescence immunohistochemical assay is qualitative at this stage, but it is possible to define its ability to discriminate between oxygenated and hypoxic cells by reference to autoradiographic studies of [$^3$H]-CCI-103F bound to spheroids labelled in air or in the absence of oxygen. The patterns of fluorescence in the spheroid sections (Figure 3) are comparable to the distribution of grain counts in EMT6/Ed spheroids grown under similar conditions. For example, a four-fold increase in [$^3$H]-CCI-103F binding occurs in going from the well-aerated outer cells to the hypoxic cells adjacent to the necrotic centre of spheroids labelled in air with [$^3$H]-CCI-103F (Figure 4, dashed line). In the case of spheroids labelled in the absence of oxygen, uniform grain counts are observed from the periphery inward to the necrotic zone (Figure 4, solid line). It can be seen (Figure 4) that spheroids labelled in air with [$^3$H]-misonidazole show a greater difference in grain density between aerobic and hypoxic cells than is the case for labelling with [$^3$H]-CCI-103F. This is because the more hydrophobic CCI-103F (Raleigh *et al.*, 1986) binds more avidly to aerated cells than does misonidazole. Nevertheless, CCI-103F binding can discriminate between these two types of cells almost as well as can misonidazole.

Figure 3c is a haematoxylin-eosin-stained section of Walker 256 carcinoma labelled *in vivo* with CCI-103F as described in **Materials and methods**. In the centre of the field a zone of relatively healthy cells is evident which is bounded on three sides by necrotic cells and cellular debris. An adjacent tissue section, immunohistochemically stained for the hypoxic marker (Figure 3d) reveals positive staining of the zone of necrotic cells and several layers of healthy cells adjacent to the necrotic regions. The fluorescent labelling of areas bordering necrotic regions was observed consistently throughout the tumour tissue sections.

### Discussion

Recent success in the use of non-invasive $^{19}$F magnetic resonance spectroscopy to detect the binding of fluorinated 2-nitroimidazoles to tumour hypoxia *in vivo* (Raleigh *et al.*, 1986; and in preparation) has led to an interest in developing an ancillary, non-radioactive histochemical assay for hypoxia. It was known that the side chain of a substituted 2-nitroimidazole is efficiently bound to hypoxic cells (Raleigh *et al.*, 1985; Rasey *et al.*, 1985*a*) and the hexafluoroisopropoxy group in CCI-103F seemed a likely target for the development of a fluorescence immunohistochemical assay. Considerable progress has been made in studies of the chemistry of the reductive activation of 2-nitroimidazoles (Whillans & Whitmore, 1981; Varghese, 1983; Raleigh &

**Figure 3** (a) Indirect immunofluorescence photomicrograph of a section of an EMT6/Ed spheroid which was labelled with CCI-103F in nitrogen. The outer surface of the spheroid is to the left. The outer rim of viable cells (approximately one-half of the field) is strongly labelled; indicating reductive binding of CCI-103F to macromolecules in the hypoxic cells. The necrotic cells and debris at the centre of the spheroid remain unlabelled ( × 500). (b) Indirect immunofluorescence photomicrograph of a section of an EMT6/Ed spheroid which was labelled with CCI-103F in air. The presence of oxygen at the spheroid periphery inhibits binding of the compound. The innermost viable cells at the edge of the necrotic centre are known to be hypoxic under the conditions of incubation with CCI-103F (normal growth conditions), and these are the cells which are strongly labelled. Cells and debris in the necrotic interior remain unlabelled ( × 500). (c) Haematoxylin-eosin stained section of Walker 256 carcinoma. Note central zone of relatively healthy cells flanked by a region of necrotic cells and connective tissue ( × 500). (d) Adjacent tissue section of Walker 256 carcinoma immunohistochemically stained for the presence of adducts of CCI-103F. The cells of normal histological appearance are unstained; regions of necrosis appear strongly fluorescent ( × 500).

**Figure 4** Grain density in autroradiograms of EMT6/Ed spheroids labelled with [³H]-misonidazole or [³H]-CCF-103F in normal growth conditions (air) or in the absence of oxygen ($N_2$). Points are the means of counts along 20 radii from ten spheroids. Error bars are 95% confidence limits. Spheroid diameters were 1.1 to 1.3 mm.

Liu, 1984; McClelland *et al.*, 1984, 1985). *In vitro* studies (Raleigh *et al.*, 1985; Rasey *et al.*, 1985*a*) appear to confirm the idea that the binding agent is a molecular product incorporating both ring and side chain of reductively-activated 2-nitroimidazoles. At present, the evidence favours an hydroxylamine derivative (II) as the binding agent (Varghese, 1983; McClelland *et al.*, 1984, 1985). The hydroxylamine intermediate is stabilised to hydrolytic decomposition at pH values less than 4 (McClelland *et al.*, 1984). This, combined with the knowledge that proteins are major sites of binding in hypoxic cells (Miller *et al.*, 1982; Smith, 1984), led to preparation of an immunogen formed by the radiation chemical reduction of CCI-103F at pH 3 in the presence of a suitable protein such as haemocyanin. The efficient binding of CCI-103F under these conditions, in a way in which the side-chain is preserved, is consistent with the binding of an hydroxylamine intermediate to protein sulphhydryl and amino groups. The situation is analogous to that proposed for the binding of reductively-activated 2-nitroimidazoles to glutathione (Varghese, 1983; Smith & Born, 1984). The successful development of a fluorescence immunohistochemical detection for CCI-103F opens the possibility that the present approach may be useful in raising antibodies to 2-nitroimidazoles with a variety of side-chains.

The hydrolytic instability of the hydroxylamine intermediate at neutral pH (McClelland *et al.*, 1984) may account for the limited diffusion of the binding agent away from the hypoxic cells (Franko *et al.*, 1982; Chapman *et al.*, 1983; Franko & Koch, 1984). This is an essential property of hypoxia markers based on the reductive metabolism of nitroaromatic compounds. The close correspondence of binding patterns of [$^{3}$H]-misonidazole and [$^{3}$H]-CCI-103F in spheroids (Figure 4) indicates that the binding intermediate formed from each has similar diffusion properties, even though the side-chain differs markedly in polarity. Nevertheless, in some cases, the side chain does affect the efficiency of reductive binding of 2-nitroimidazoles to both cells (Chapman *et al.*, 1983) and nucleic acids (Silver *et al.*, 1986). These distinctions may ultimately be important to hypoxia marker development.

The fluorescent labelling pattern observed in oxygenated and hypoxic spheroids is consistent with the pattern previously demonstrated by autoradiography (Figure 4 and Franko *et al.*, 1982, 1984; Franko, 1985, 1986). The labelling pattern of cells adjacent to necrotic regions in the Walker 256 carcinoma is consistent with the hypoxic fraction of 7% reported for this tumour (Clement *et al.*, 1978), and is encouraging regarding the utility of the immunohistochemical approach to detect hypoxia *in vivo*. The amount of CCI-103F used to label the tumour *in vivo* produced no obvious long term toxicity although a transient depressive effect was noted. Lower whole body concentrations over longer exposure times, which was achieved by repeated injections, also produced binding of CCI-103F to hypoxic cells in tumours but no obvious long term toxicity (Raleigh *et al.*, 1986). A much more detailed study of CCI-103F toxicity is required, however, before its clinical application is considered.

The long range goal of our research is to develop a physically non-invasive method of measuring hypoxia in human tumours. We believe that $^{19}$F MRS offers some potential in this regard (Raleigh *et al.*, 1986; and in preparation) and are studying a selection of fluorinated 2-nitroimidazoles as potential markers. The availability of a correlative assay during the development stages of the $^{19}$F MRS approach is viewed as essential. To date we have relied on quantitative autoradiography and scintillation counting of excised tumours labelled with tritiated versions of the fluorinated compounds as a means of establishing the $^{19}$F MRS technique. The fluorescence immunohistochemical technique would have the important advantage of eliminating the need for using radioactive compounds if the fluorescence intensity could be quantified. Efforts to do so are presently underway.

It is conceivable that in the absence of a physically non-invasive assay of tumour hypoxia such as $^{19}$F MRS, a fluorescence immunohistochemical technique applied to biopsy material could become an acceptable way of establishing the degree of initial tumour hypoxia or, with multiple fluorescence agents, the course of reoxygenation during radiation therapy. In fact, even with a non-invasive technique like $^{19}$F MRS which can only provide an integrated signal of total drug binding to a tumour, it may be necessary to establish by means of an histochemical examination of a biopsy sample what the initial distribution of hypoxia is in the tumour. There would be a real advantage to using a non-radioactive marker for this step. It is also clear that a fluorescence immunohistochemical assay based on 2-nitroimidazoles which are presently in clinical use is desirable and attempts to develop such an assay have been undertaken.

The authors thank Dr Janet Sharplin and Ms Ruth Sutherland for excellent technical assistance, Ms Jan Spivak-Steele for histological preparations, Ms Gina Kennedy for assistance in preparing the manuscript and Dr Andrew Shaw for use of the EL 309 Microplate Autoreader (Bio-Tek Instruments). The authors are pleased to acknowledge the Alberta Cancer Board, the Alberta Heritage Savings Trust Fund – Applied Research Cancer and the National Cancer Institute of Canada for financial support.

### References

BORN, J.L. & SMITH, B.R. (1983). The synthesis of tritium-labelled misonidazole. *J. Labelled Compounds Radiopharmaceut.*, **xx**, 429.

CHAPMAN, J.D. (1984). The detection and measurement of hypoxic cells in solid tumours. *Cancer*, **54**, 2441.

CHAPMAN, J.D., BAER, K. & LEE, J. (1983). Characteristics of the metabolism-induced binding of misonidazole to hypoxic mammalian cells. *Cancer Res.*, **43**, 1523.

CHAPMAN, J.D., FRANKO, A.J. & SHARPLIN, J. (1981). A marker for hypoxic cells in tumours with potential clinical applicability. *Br. J. Cancer*, **43**, 546.

CLEMENT, J.J., TANAKA, N. & SONG, C.W. (1978). Tumor reoxygenation and postirradiation vascular changes. *Radiology*, **127**, 799.

FRANKO, A.J. (1985). Hypoxic fraction and binding of misonidazole in EMT6/Ed multicellular tumour spheroids. *Radiat. Res.*, **103**, 89.

FRANKO, A.J. (1986). Misonidazole and other hypoxia markers: metabolism and applications. *Int. J. Radiat. Oncol.*, **12**, 1195.

FRANKO, A.J., CHAPMAN, J.D. & KOCH, C.J. (1982). Binding of misonidazole to EMT6 and V79 spheroids. *Int. J. Radiat. Oncol. Biol. Phys.*, **8**, 737.

FRANKO, A.J., FREEDMAN, H.I. & KOCH, C.J. (1984). Oxygen supply to spheroids in liquid overlay and spinner culture. *Recent Res. Cancer Res.*, **95**, 162.

FRANKO, A.J. & KOCH, C.J. (1983). The radiation response of hypoxic cells in EMT6 spheroids in suspension culture does model data from EMT6 tumors. *Radiat. Res.*, **96**, 497.

FRANKO, A.J. & KOCH, C.J. (1984). Binding of misonidazole to V79 spheroids and human colon carcinomas *in vitro*: Diffusion of oxygen and reactive metabolites. *Int. J. Radiat. Oncol. Biol. Phys.*, **10**, 1333.

FUCIARELLI, A.F., MILLER, G.G. & RALEIGH, J.A. (1985). An immunochemical probe for 8,5′-cycloadenosine 5′-monophosphate and its deoxy analogue in irradiated nucleic acids. *Radiat. Res.*, **104**, 272.

GARRECHT, B.M. & CHAPMAN, J.D. (1983). The labelling of EMT-6 tumours in Balb/C mice with $^{14}$C-misonidazole. *Br. J. Radiol.*, **56**, 745.

HIRST, D.G., HAZLEHURST, J.L. & BROWN, J.M. (1985). Changes in misonidazole binding with hypoxic fraction in mouse tumours. *Int. J. Radiat. Oncol. Biol. Phys.*, **11**, 1349.

JETTE, D.C., WIEBE, L.I. & CHAPMAN, J.D. (1983). Synthesis and *in vivo* studies of the radiosensitizer 4-[$^{82}$Br] bromomisonidazole. *Int. J. Nucl. Med. Biol.*, **10**, 205.

KENNEDY, K.A., TEICHER, B.A., ROCKWELL, S. & SARTORELLI, A.C. (1980). The hypoxic tumour cell: A target for selective cancer chemotherapy. *Biochem. Pharmacol.*, **29**, 1.

McCALLA, D.R., REUVERS, A. & KAISER, C. (1970). Mode of action of nitrofurazone. *J. Bacteriol.*, **104**, 1126.

McCLELLAND, R.A., FULLER, J.R. & SEAMAN, N.E. (1984). 2-Hydroxylaminoimidazoles – unstable intermediates in the reduction of 2-nitroimidazoles. *Biochem. Pharmacol.*, **33**, 303.

McCLELLAND, R.A., PANICUCCI, R. & RAUTH, A.M. (1985). Electrophilic intermediate in the ractions of a 2-(hydroxylamino) imidazoles. A model for biological effects of reduced nitroimidazoles. *J. Amer. Chem. Soc.*, **107**, 1762.

MILLER, G.G., NGAN-LEE, J. & CHAPMAN, J.D. (1982). Intracellular localization of radioactively-labelled misonidazole in EMT6 tumour cells *in vitro*. *Int. J. Radiat. Oncol. Biol. Phys.*, **8**, 741.

RALEIGH, J.A., FRANKO, A.J., KOCH, C.J. & BORN, J.A. (1985). Binding of misonidazole to hypoxic cells in monolayer and spheroid culture. Evidence that a side-chain label is bound as efficiently as a ring label. *Br. J. Cancer*, **51**, 229.

RALEIGH, J.A., FRANKO, A.J., TREIBER, E.O., LUNT, J.A. & ALLEN, P.S. (1986). Covalent binding of a fluorinated 2-nitroimidazole to EMT-6 tumours in Balb/C mice: Detection by F-19 nuclear magnetic resonance at 2.35 T. *Int. J. Radiat. Oncol. Biol. Phys.*, **12**, 1243.

RALEIGH, J.A. & LIU, S.F. (1984). Reductive fragmentation of 2-nitroimidazoles: Amines and aldehydes. *Int. J. Radiat. Oncol. Biol. Phys.*, **10**, 1337.

RASEY, J.S., GRUNBAUM, Z., KROHN, K., NELSON, N. & CHIU, L. (1985*a*). Comparison of binding of [$^{3}$H]misonidazole and [$^{14}$C]misonidazole in multicellular spheroids. *Radiat. Res.*, **101**, 473.

RASEY, J.S., KROHN, K.A., GRUNBAUM, Z., CONROY, P.J., BAUER, K. & SUTHERLAND, R.M. (1985*b*). Further characterization of 4-bromomisonidazole as a potential detector of hypoxic cells. *Radiat. Res.*, **102,** 76.

SAINTE-MARIE, G. (1962). A paraffin embedding technique for studies employing immunofluorescence. *J. Histochem. Cytochem.*, **10,** 250.

SILVER, A.R.J., McNEIL, S.S., O'NEIL, P., JENKINS, T.C. & AHMED, I. (1986). Induction of DNA strand breaks by reduced nitro-imidazoles. Implications for DNA base damage. *Biochem. Pharmacol.*, **35,** 3923.

SMITH, B.R. (1984). Hypoxia-enhanced reduction and covalent binding of 2-[$^3$H]-misonidazole in the perfused rat liver. *Biochem. Pharmacol.*, **33,** 1379.

SMITH, B.R. & BORN, J.L. (1984). Metabolism and excretion of [$^3$H] misonidazole by hypoxic rat liver. *Int. J. Radiat. Oncol. Biol. Phys.*, **10,** 1365.

URTASUN, R.C., CHAPMAN, J.D., RALEIGH, J.A., FRANKO, A.J. & KOCH, C.J. (1986*a*). Binding of $^3$H-misonidazole to solid human tumours as a measure of tumour hypoxia. *Int. J. Radiat. Oncol. Biol. Phys.*, **12,** 1263.

URTASUN, R.C., KOCH, C.J., FRANKO, A.J., RALEIGH, J.A. & CHAPMAN, J.D. (1986*b*). A novel technique for measuring human tissue $pO_2$ at the cellular level. *Br. J. Cancer*, **54,** 453.

VARGHESE, A.J. (1983). Glutathione conjugates of misonidazole. *Biochem. Biophys. Res. Commun.*, **112,** 1013.

VARGHESE, A.J., GULYAS, S. & MOHINDRA, J.K. (1976). Hypoxia-dependent reduction of 1-(2-nitro-1-imidazoyl)-3-methoxy-2-propanol by Chinese hamster ovary cells and KHT tumour cells *in vitro* and *in vivo*. *Cancer Res.*, **36,** 3761.

WHILLANS, D.W. & WHITMORE, G.F. (1981). The radiation reduction of misonidazole. *Radiat. Res.*, **86,** 311.

WIEBE, L.I., JETTE, D.C. & CHAPMAN, J.D. (1984). Electron-affinic compounds for labelling hypoxic cells: The synthesis and characterization of 1-[2-(2-iodophenoxy)-ethyl]-2-nitroimidazole. *Nuclearmedizin*, **23,** 63.